# Use of Iodine-Containing Dietary Supplements Remains Low among Women of Reproductive Age in the United States: NHANES 2011–2014

**DOI:** 10.3390/nu10040422

**Published:** 2018-03-29

**Authors:** Priya M. Gupta, Jaime J. Gahche, Kirsten A. Herrick, Abby G. Ershow, Nancy Potischman, Cria G. Perrine

**Affiliations:** 1 Division of Nutrition, Physical Activity, and Obesity, Centers for Disease Control and Prevention, Atlanta, GA 30341, USA; cperrine@cdc.gov; 2 National Institutes of Health, Office of Dietary Supplements, Bethesda, MD 20892, USA; jaime.gahche@nih.gov (J.J.G.); ershowa@od.nih.gov (A.G.E.); potischn@mail.nih.gov (N.P.); 3 National Center for Health Statistics, Centers for Disease Control and Prevention, Hyattsville, MD 20782, USA; kherrick1@cdc.gov

**Keywords:** iodine, supplements, pregnant, lactating, women of reproductive age

## Abstract

In the United States, the American Thyroid Association recommends that women take a dietary supplement containing 150 µg of iodine 3 months prior to conception and while pregnant and lactating to support fetal growth and neurological development. We used data from the National Health and Nutrition Examination Survey 2011–2014 to describe the use of dietary supplements with and without iodine in the past 30 days among 2155 non-pregnant, non-lactating (NPNL) women; 122 pregnant women; and 61 lactating women. Among NPNL women, 45.3% (95% Confidence Interval [CI]: 42.0, 48.6) used any dietary supplement and 14.8% (95% CI: 12.7, 16.8) used a dietary supplement with iodine in the past 30 days. Non-Hispanic black and Hispanic women were less likely to use any dietary supplement as well as one with iodine, than non-Hispanic white or non-Hispanic Asian women (*p* < 0.05). Among pregnant women, 72.2% (95% CI: 65.8, 78.6) used any dietary supplement; however, only 17.8% (95% CI: 11.4, 24.3) used a dietary supplement with iodine. Among lactating women, 75.0% (95% CI: 63.0, 87.0) used a dietary supplement; however, only 19.0% (95% CI: 8.8, 29.2) used a dietary supplement with iodine. Among NPNL women using a supplement with iodine, median daily iodine intake was 75.0 µg. Self-reported data suggests that the use of iodine containing dietary supplements among pregnant and lactating women remains low in contrast with current recommendations.

## 1. Introduction

Iodine is an essential component of the thyroid hormones, thyroxine and triiodothyronine. These hormones regulate vital body functions including fetal and postnatal growth and neurologic development [1]. During pregnancy, iodine requirements increase to accommodate fetal needs and alterations in maternal iodine metabolism, including increased urinary iodine loss [2,3]. Adequate iodine intake is also critical early in pregnancy when the fetal brain is growing rapidly. Studies have shown that overt iodine deficiency is associated with irreversible neurological damage in the fetus [3]. Other studies suggest that even mild iodine deficiency is associated with poor cognitive development and educational attainment in young children [4,5].

The recommended daily allowance (RDA) of iodine for non-pregnant, non-lactating women (NPNL) is 150 µg/day and increases to 220 µg/day and 290 µg/day for pregnant and lactating women, respectively [6]. Given the increased demand for iodine during pregnancy and lactation, the high variability of iodine in foods [7,8,9,10] and the limited exposure to iodine from fortified salt [11], dietary intake alone may not be sufficient to ensure optimal iodine nutrition [8,12]. Therefore, it is important to monitor the use of dietary supplements containing iodine among women of reproductive age, as iodine is a nutrient critical for fetal brain development, and pregnant women in the US, as a group, may be iodine deficient [13].

In 2006, the American Thyroid Association (ATA) recommended that pregnant and lactating women in the United States and Canada take a dietary supplement containing 150 µg of iodine [14]. In 2014, the American Academy of Pediatrics (AAP) released a similar recommendation, advocating the use of an iodine supplement during pregnancy [15]. In 2017, the ATA added that women who are planning pregnancy should begin taking a supplement with iodine three months in advance of the planned pregnancy [16]. The last national estimates of iodine containing dietary supplement usage among women of reproductive age used data up to 2006 [17,18]. This study provides updated estimates of the prevalence of dietary supplement use (any or iodine-containing) among women of reproductive age (20–44 years, including pregnant and lactating women) in the United States.

## 2. Materials and Methods

### 2.1. Survey Design

The National Health and Nutrition Examination Survey (NHANES) is an ongoing, nationally representative survey of the civilian, non-institutionalized population in the United States. NHANES uses a complex, stratified, multistage probability cluster sampling design. Detailed information on the study design and methods is available elsewhere [19,20]. Briefly, data collection in NHANES includes a household interview and a physical examination conducted in the Mobile Examination Center (MEC). Informed consent was obtained from all adult participants, and the sampling protocol was approved by National Center for Health Statistics Research Ethics Review Board [20].

### 2.2. Sample Selection

To improve the reliability and stability of estimates, we pooled data from two cycles of NHANES: 2011–2012 and 2013–2014 (examination response rates for women were 69.4% and 68.8%, respectively) [21]. Pregnancy status was determined in the MEC. The MEC sample included 2517 women aged 20–44 years. Pregnancy status of adolescents (aged < 20 years) is not available in the publicly available NHANES data files; therefore, we limited our sample to adult women. Women were excluded if pregnancy status could not be ascertained (*n* = 179). The remaining 2338 women were further categorized into pregnant, lactating, and NPNL. Pregnant women were defined as those with a positive pregnancy test or self-reported pregnancy. Lactating women were those who reported that they were currently breastfeeding. NPNL women were defined as those who were not pregnant and did not report current breastfeeding. Two women were both pregnant and lactating; these women were included with the pregnant group only. Conceptually, we felt that pregnancy would drive supplement use more than lactation. We do not anticipate our results would change significantly, given there were only two women who were both pregnant and lactating. Our final analytic sample included 2338 women, of which 2155, 122, and 61 were NPNL, pregnant, and lactating, respectively.

### 2.3. Demographic Variables

Age was categorized into 5-year intervals: 20–24 years, 25–29 years, 30–34 years, 35–39 years, and 40–44 years. Self-reported race/Hispanic origin was categorized into: non-Hispanic white, non-Hispanic black, non-Hispanic Asian, and Hispanic. NHANES participants reporting ‘other’ race or multiple races are not shown separately, but are included in the overall and estimates for other demographic categorizations. Federal income to poverty ratio, calculated by dividing family income by the US Department of Health and Human Services poverty guidelines specific to the survey year, was categorized as follows: 0–185%, >185–350%, and >350% (based on WIC eligibility criteria) [22].

### 2.4. Dietary Supplement Data

Dietary supplement users included those who reported using any dietary supplement in the past 30 days, whereas iodine-containing supplement users were those that reported using a dietary supplement with iodine in the past 30 days. Data on dietary supplement use in the previous 30 days were collected during the household interview. Participants were asked about their usage of vitamins, minerals, herbals, and other dietary supplements; this included prescription and non-prescription products. Participants were then asked to show interviewers the containers for all products taken so that information from the label could be recorded, including product name and manufacturer or distributor name and address. Data were obtained from product labels post-interview for information on serving size, nutrients, and nutrient amounts. Participants were also asked about the frequency of use and amount typically used.

Descriptive statistics of daily iodine intake from supplements were derived among women who reported using a supplement with iodine (*n* = 323). The mean daily intakes of iodine reported in the NHANES total supplement files were pre-calculated by dividing the amount of iodine (based on the serving size listed on the product label) by the number of days the supplement had been used in the past 30 days. These methods are described in detail elsewhere [23,24]. Among iodine supplement users, frequency of iodine supplement use was categorized as 1–7, 8–15, 16–29, and 30 days.

### 2.5. Statistical Analyses

All statistical analyses were performed using SAS-Callable SUDAAN (version 11.0.1) software. MEC weights were used to account for NHANES’s complex survey design (including oversampling) and survey non-response. Weighted prevalence estimates for use of at least one dietary supplement in the past 30 days and use of a dietary supplement containing iodine were calculated for all women. Prevalence estimates for all women and NPNL women were further stratified by age, race/Hispanic origin and family income to poverty ratio. Some sample sizes varied due to missing data on covariates, and this is indicated in table footnotes. Variance estimates for all statistics of interest were approximated by Taylor Series Linearization, accounting for the complex design of NHANES. Differences between groups were evaluated using a t statistic. Tests of linear trend across ordinal variables were evaluated using orthogonal contrast matrices. Statistical significance was set as *p* < 0.05. Estimates with a relative standard error ([(standard error of the prevalence/prevalence) * 100]) <30% are presented [25]. Stratified data for pregnant and lactating women are not presented due to small sample sizes. We did not account for multiple comparisons.

## 3. Results

### 3.1. All Women

Approximately half of women (47.6%; 95% CI: 44.3, 50.9) in our sample (including pregnant and lactating women) had used a dietary supplement in the past 30 days; however, 15.1% (95% CI: 13.2, 16.9) had used a dietary supplement containing iodine (Table 1). Younger women (aged 20–24 years) were significantly less likely to take any dietary supplements than women aged ≥25 years. Use of both any supplement and use of iodine-containing dietary supplements varied by race/Hispanic origin. For example, the prevalence of using any dietary supplement was significantly lower among non-Hispanic black women (35.3%; 95% CI: 30.6, 39.9) and Hispanic women (39.3%; 95% CI: 34.8, 43.9) compared to non-Hispanic white women (53.1%; 95% CI: 48.6, 57.5) and non-Hispanic Asian women (52.0%; 95% CI: 46.3, 57.7) women. In addition, the prevalence of using an iodine-containing dietary supplement was significantly lower among non-Hispanic black women (10.9%; 95% CI: 8.8, 13.1) as compared to non-Hispanic white women (17.1%; 95% CI: 14.0, 20.1) and non-Hispanic Asian women (14.4%; 95% CI: 11.8, 17.0) but did not significantly differ from that among Hispanic women (13.5%; 95% CI: 10.0, 17.0). Supplement use increased with income (*p* < 0.05 for linear trend). Women at or below 185% of the federal poverty level had a lower prevalence of any or iodine containing dietary supplement use as compared to women above 185% of the federal poverty level. A higher percentage of women with greater than a high school degree reported taking any supplement or iodine-containing dietary supplements than women with less than a high school degree.

### 3.2. Non-Pregnant, Non-Lactating Women

Less than half of NPNL women (45.3%; 95% CI: 42.0, 48.6) reported the use of a dietary supplement, and 14.8% (95% CI: 12.7, 16.8) reported the use of a dietary supplement containing iodine. A higher percentage of older women (aged 40–44 years) reported taking a dietary supplement (any or iodine-containing) as compared to younger women (aged 20–24 years) (*p* < 0.05). Non-Hispanic black and Hispanic women were significantly less likely to use a dietary supplement (any or iodine-containing) than non-Hispanic white or non-Hispanic Asian women. Prevalence estimates of dietary supplement use by federal income to poverty ratio and education level were similar to that of all women (*p* < 0.05 for linear trend for income and education level).

### 3.3. Pregnant Women

Although 72.2% (95% CI: 65.8, 78.6) of pregnant women reported using any dietary supplement, 17.8% (95% CI: 11.4, 24.3) reported using a dietary supplement with iodine.

### 3.4. Lactating Women

Among lactating women, 75.0% (95% CI: 63.0, 87.0) reported using a dietary supplement; however, 19.0% (95% CI: 8.8, 29.2) reported using a dietary supplement with iodine.

### 3.5. Daily Iodine Intake from Iodine-Containing Dietary Supplements

Among all women who reported using a dietary supplement with iodine, the daily intake of iodine from dietary supplements was right skewed. Median daily intake of iodine from dietary supplements was 75.0 µg among both all women and NPNL women (Table 2). Results for pregnant and lactating women are not shown due to small sample size.

Half of all women who reported using a dietary supplement with iodine (*n* = 323) used the dietary supplement for the full 30 days. The percentages of women who reported using a dietary supplement with iodine for 1–7 days, 8–15 days, and 12–29 days were 15.1%, 18.6% and 16.4%, respectively (data not shown).

## 4. Discussion

About one in seven women of reproductive age (15.1%), including pregnant and lactating women, reported taking a dietary supplement that contained iodine. Two previous analyses of NHANES (1999–2006 and 2001–2006) described the prevalence of iodine-containing dietary supplement use among women of reproductive age [17,18]. At that time, approximately 20% of pregnant and non-pregnant women, and 15% of lactating women, were taking a dietary supplement that contained iodine. Our data suggest there has been little change since that time (17.8% pregnant and 19.0% lactating). 

In 1999–2006, the median daily intake among women aged 15–39 years (including pregnant but not lactating) who reported taking a dietary supplement containing iodine was 124 µg/day [17]. This is higher than the 75 µg/day reported here, suggesting iodine intake among iodine supplement users may be lower than in previous years. While NHANES did oversample pregnant women in 1999–2006, the median iodine intake from supplements among NPNL women was 112 ug/day, compared to 75 ug/day for NPNL women in 2011–2014. This suggests that sample composition of the two study periods is not driving the difference in iodine intake from supplements.

It is important to note that both of these estimates are based on a 30-day frequency questionnaire. Therefore average daily nutrient intake over 30 days is calculated using information on the frequency of use, servings consumed, and iodine content in the serving (based on the product label); changes in median consumption can reflect changes in any of these three pieces of information. Our study found that 73.9% of women taking a supplement containing iodine were taking a product with at least 150 µg per serving (data not shown). Saldanha et al. found that for dietary supplements that were specifically marketed as prenatal supplements, the mean iodine content of prescription prenatal supplements was 150 ± 4.8 µg and for non-prescription prenatal supplements, it was 164 ± 6.7 ug [26]. As it is unlikely there has been a dramatic reduction in the amount of iodine included in dietary supplements containing at least some iodine, the lower median may be due to women reporting less frequent use of supplements. Data from the 1999–2006 NHANES indicated that 65% of women who reported using a dietary supplement with iodine used the supplement daily (unpublished data, Jaime Gahche, NIH). We found that only half of all women who reported using a dietary supplement with iodine used the dietary supplement daily, suggesting that frequency of iodine containing supplement use may be declining.

In 2014, the AAP released a recommendation for pregnant and lactating women to take a dietary supplement containing iodine [15], reinforcing the 2006 recommendation from ATA. While the majority of pregnant and lactating women in the current study were taking a supplement, our analysis suggests that many of the supplements consumed by these women do not contain iodine. An analysis in 2009 found that 51% of prenatal vitamins marketed in the US contained iodine [27], while a study published in 2017 found that 61% of commercially available prenatal vitamins in the US contained iodine [28]. Some of the increase in the inclusion of iodine in prenatal vitamins may be due to increased recognition by the supplement industry of the recommendations by various health agencies for iodine supplementation during pregnancy and lactation [28].

This study has several strengths. We used data from the NHANES, a nationally representative data source. We presented national estimates and estimates for various subsets of the US population, including the first nationally representative estimates for non-Hispanic Asian women. This study is also subject to limitations. First, the NHANES dietary supplement database relies on the manufacturers’ labels to determine the amount of iodine in the products. The nutrient content of a dietary supplement can vary from what is reported on the nutrition label. A 2017 study by Andrews et al. found that the reported iodine content of a supplement can exceed the amount reported on the label by an average of 20.2% [29]. Second, we currently lack the appropriate database necessary for estimating the iodine intake from dietary sources; therefore, we are unable to estimate how much iodine supplements add to daily intakes. Currently, there is effort being taken in the US to develop a dietary database for iodine [30]. Third, we were limited by the small number of pregnant and lactating women. As a result, we were not able to produce estimates of the prevalence of dietary supplement use and iodine-containing dietary supplement use by sociodemographic characteristics among pregnant and lactating women.

In many countries, diet alone is not sufficient to meet the increased demand for iodine during pregnancy and lactation [8,12], and supplements may be an important source of additional iodine for these groups. Thus, it is necessary to monitor the use of dietary supplements containing iodine among key target groups.

## Figures and Tables

**Table 1:** Prevalence of supplement use and iodine-containing supplement use among women of reproductive age (20–44 years) by demographic stratifications: NHANES 2011–2014.

	*n*	% Using Any Dietary Supplement ^1^	95% Confidence Interval	% Using a Dietary Supplement with Iodine ^1^	95% Confidence Interval
**All women**	2338	47.6	(44.3, 50.9)	15.1	(13.2, 16.9)
Age (years) ^2^					
20–24	473	39.4 ^a^	(32.6, 46.2)	10.9 ^a^	(7.3, 14.5)
25–29	419	47.0 ^b^	(40.9, 53.1)	16.1 ^a,b^	(11.1, 21.1)
30–34	464	48.9 ^b^	(43.0, 54.9)	16.2 ^a,b^	(12.5, 19.9)
35–39	466	50.4 ^b^	(43.5, 57.3)	14.4 ^a,b^	(10.0, 18.8)
40–44	516	52.6 ^b^	(46.9, 58.3)	18.0 ^b^	(13.7, 22.3)
Race/Hispanic Origin ^4^					
Non-Hispanic White	834	53.1 ^a^	(48.6, 57.5)	17.1 ^a^	(14.0, 20.1)
Non-Hispanic Black	533	35.3 ^b^	(30.6, 39.9)	10.9 ^b^	(8.8, 13.1)
Non-Hispanic Asian	343	52.0 ^a^	(46.3, 57.7)	14.4 ^a^	(11.8, 17.0)
All Hispanic	314	39.3 ^b^	(34.8, 43.9)	13.5 ^a,b^	(10.0, 17.0)
Poverty to Income Ratio ^3,5^					
0–185%	1146	39.6 ^a^	(35.0, 44.2)	9.9 ^a^	(7.8, 12.1)
>185–350%	445	50.5 ^b^	(43.8, 57.3)	16.9 ^b^	(13.7, 20.0)
>350%	599	57.6 ^b^	(51.7, 63.6)	20.4 ^b^	(15.3, 25.5)
Education level ^3,5^					
<High school degree	375	32.1 ^a^	(27.4, 36.8)	8.3 ^a^	(5.4, 11.2)
High school degree	411	42.6 ^b^	(37.4, 47.8)	11.9 ^a^	(8.4, 15.4)
>High school degree	1550	51.7 ^c^	(47.5, 55.9)	17.1 ^b^	(14.4, 19.8)
**Non-pregnant, non-lactating women**	2155	45.3	(42.0, 48.6)	14.8	(12.7, 16.8)
Age (years) ^2^					
20–24	424	37.6 ^a^	(30.1, 45.2)	11.1 ^a^	(7.1, 15.2)
25–29	376	43.7 ^a^	(37.2, 50.2)	16.6 ^a,b^	(11.1, 22.1)
30–34	413	43.9 ^a^	(38.4, 49.4)	14.8 ^a,b^	(11, 18.7)
35–39	438	48.4 ^a,b^	(41.2, 55.6)	13.3 ^a,b^	(8.5, 18.0)
40–44	504	52.2 ^b^	(46.4, 58.1)	18.2 ^b^	(13.8, 22.6)
Race/Hispanic Origin ^4^					
Non-Hispanic White	762	50.2 ^a^	(45.5, 54.9)	16.6 ^a^	(13.2, 19.9)
Non-Hispanic Black	489	34.7 ^b^	(29.4, 40.0)	11.5 ^b^	(9.3, 13.7)
Non-Hispanic Asian	317	50.2 ^a^	(44.3, 56)	13.9 ^a^	(11.0, 16.8)
All Hispanic	295	37.4 ^b^	(32.9, 42)	13.2 ^b^	(9.6, 16.6)
Poverty to Income Ratio ^3,5^					
0–185%	1056	37.5 ^a^	(32.9, 42.1)	10.1 ^a^	(8.0, 12.2)
>185–350%	409	48.9 ^b^	(42.1, 55.7)	16.3 ^b^	(12.8, 19.9)
>350%	552	55.1 ^b^	(48.6, 61.5)	20.2 ^b^	(14.7, 25.7)
Education level ^3,5^					
<High school degree	348	29.7 ^a^	(24.6, 34.8)	8.7 ^a^	(5.7, 11.8)
High school degree	384	41.4 ^b^	(36.4, 46.4)	12.0 ^a,b^	(8.3, 15.7)
>High school degree	1421	49.2 ^c^	(45.1, 53.3)	16.7 ^b^	(13.7, 19.6)
**Pregnant women**	122	72.2	(65.8, 78.6)	17.8	(11.4, 24.3)
**Lactating women**	61	75.0	(63.0, 87.0)	19.0	(8.8, 29.2)

^1^ All analyses were weighted and took into account the complex survey design. Estimates that share the same superscript do not significantly differ from one another (significance is based on *t*-test values of *p* < 0.05). ^2^ Linear trend in any supplement use. ^3^ Linear trend in any supplement use and iodine-containing supplement use. ^4^ Other race/Hispanic origin is included in totals but not shown separately. ^5^ Sample sizes vary due to missing data.

**Table 2:** Daily intake of iodine from supplements containing iodine among women of reproductive age (20–44 years): NHANES 2011–2014 ^1^

	*n*	Mean	95% Confidence Interval	Median	Interquartile Range
All women	323	88.3	(80.6, 96.1)	75.0	(113.0)
Non-pregnant, non-lactating women	295	87.8	(80.1, 95.5)	75.0	(114.0)

^1^ Estimates of iodine intake are only among users of iodine-containing dietary supplements.
